# Improving the recognition of child maltreatment in emergency departments in Europe: healthcare professionals’ perceived barriers and facilitators for implementation of a comprehensive toolkit design

**DOI:** 10.1007/s00431-026-06815-8

**Published:** 2026-04-08

**Authors:** F. Hoedeman, P. J. Puiman, A. W. Smits, M. I. Dekker, D. Lauwaert, R. Oostenbrink, N. Parri, L. García-Castrillo Riesgo, S. Polinder, H. A. Moll

**Affiliations:** 1 Department of General Paediatrics, Erasmus MC-Sophia Children’s Hospital, P.O. Box 2060, 3000 CB Rotterdam, The Netherlands; 2 Augeo Foundation, Driebergen, The Netherlands; 3 Emergency Department, University Hospital Brussels, Brussels, Belgium; 4 Meyer Children’s Hospital IRCCS, Florence, Italy; 5 Emergency Department, University Hospital Marques Valdecilla, Cantabria, Spain; 6 Department of Public Health, Erasmus University Medical Centre, Rotterdam, The Netherlands

**Keywords:** Child abuse, Emergency departments, Screening, Europe

## Abstract

Strategies to improve child maltreatment recognition in emergency departments (EDs) have been associated with increased detection. However, implementing new protocols in high-pressure environments is challenging, necessitating an understanding of healthcare professionals’ perceptions. This study explored perceived barriers and facilitators for implementing a child maltreatment toolkit design in European EDs. The presented toolkit design included the Screening instrument for Child Abuse & Neglect (SCAN), training, and hospital policy. To assess barriers and facilitators for its implementation, a survey was developed using the ‘Barriers and Facilitators Assessment Instrument’ featuring statements on a 5-point Likert scale with additional open-ended questions. This pre-implementation survey was distributed to ED professionals through European emergency medicine societies and research networks (EUSEM, REPEM, and EuSEN) and additional contacts via snowball sampling. Descriptive statistics and thematic analyses of open-ended responses were conducted. A total of 204 respondents participated, representing 121 hospitals from 28 European countries (estimated response 36%–46%). Facilitators for implementation included the toolkit’s alignment with the ED environment, covering time, financial and staff resources, ED staff motivation and the hospital policy’s standardized hospital-wide implementation. Thematic analyses revealed comparable benefits, but additional disadvantages including cultural differences, risk of false positives, electronic health record (EHR) compatibility and ensuring practical over theoretical learning. *Conclusion*: This pre-implementation study highlights generally positive perceptions among European ED professionals regarding a structured child maltreatment toolkit design. Concurrently, important contextual and ethical considerations, including screening performance, legal frameworks, system capacity and potential unintended consequences, were identified. This underscores the need for careful contextual adaptation and prospective effectiveness–implementation evaluation before broader adoption.

**Table:** 

**What is Known:** • *European EDs generally lack strategies to improve the recognition of child maltreatment*. • *Successful implementation of such strategies in EDs requires an understanding of healthcare professionals’ perceived barriers and facilitators*.	
**What is New:** • *This study explores perceptions of European ED professionals regarding a child maltreatment toolkit design comprising the Screening instrument for Child Abuse and Neglect (SCAN), targeted training, and hospital policy guidance*. • *Addressing training costs, cultural variability, and concerns about false positives is essential to support implementation and to enable wider adoption, enhancing pediatric readiness for child protection across Europe*.

**Supplementary Information:**

The online version contains supplementary material available at 10.1007/s00431-026-06815-8.

## Introduction

Protecting children from all forms of harm is a core priority in Europe, as stated by the European Commission [1]. In Europe, it is estimated that 55 million children have experienced at least one type of maltreatment during childhood, which emphasizes the magnitude of this public health problem [2].

In addition, child maltreatment causes a huge socioeconomic burden with long-lasting effects for both individuals and society [3–5]. Healthcare professionals working at emergency departments (EDs) are often the first point of contact for children experiencing maltreatment, making their role pivotal in timely and effective intervention [6–8]. However, identifying child abuse cases can be complex, and child maltreatment often remains unrecognized [9].

Previous research has shown that different strategies for child maltreatment recognition are effective in increasing its detection rate, such as the use of a screening instrument [7, 10–14], recognizing parental risk factors in adult patients admitted at the ED [15, 16], training of hospital staff [11, 17–19], and a child abuse team [20–24] or policy officer [23–25]. Our previous survey amongst healthcare professionals showed only 25% of European EDs met the NICE (National Institute for Health and Care Excellence) guideline recommendations on child abuse and neglect [26], including the aforementioned strategies [27]. This calls for improvement of child maltreatment policies at the ED. Therefore, we designed a child maltreatment toolkit, including the Screening instrument for Child Abuse & Neglect (SCAN), training modules, and a strategy for establishing adequate hospital policy regarding child maltreatment for use at the ED.

Introducing new strategies in healthcare settings, particularly in high-pressure environments like EDs, presents a unique set of challenges [28]. Understanding the barriers and facilitators healthcare professionals may encounter during the implementation process is crucial for achieving effective change and ensuring sustainability of new procedures [29]. To increase the chance of successful future implementation, we developed a pre-implementation questionnaire to explore healthcare professionals’ perceived barriers and facilitators regarding the proposed child maltreatment toolkit design, including the SCAN, training, and hospital policy.

## Methods

### Toolkit child maltreatment

The design of the toolkit that was presented to healthcare professionals consisted of the following key elements (Online Resource 1):

1. Screening instrument for Child Abuse & Neglect (SCAN) [10]: a four-question screening checklist validated in the Dutch context for children aged 0 to 18 years old at the ED. The SCAN is short and easy to complete, and it is aimed to support healthcare professionals in identifying possible cases of child maltreatment. A positive screening result indicates a potential risk of abuse and necessitates a comprehensive assessment, including a full history, top-to-toe examination, additional diagnostics if necessary, and expert opinion.
2. Training modules: Since training is essential to raise awareness and knowledge about child abuse, English e-learning on recognition of signals of child maltreatment, use of the SCAN, and stress-sensitive communication were developed by the Augeo Foundation [30]. A comparable e-learning programme on child abuse from Augeo Foundation has been shown to improve performances in case simulations and nurses’ self-efficacy in the detection of child maltreatment in the ED [18].
3. Hospital policy: a written statement describing the standard of care including assessment and management of patients with suspected child maltreatment such as the presence of a child abuse team and/or policy officer, providing education, use of a (digital) screening checklist and follow-up guidelines [26, 31].

### Survey based on Barriers and Facilitators Assessment Instrument

A survey was developed to assess possible barriers and facilitators perceived by healthcare professionals at European EDs prior to implementation of this child maltreatment toolkit design. The “Barriers and Facilitators Assessment Instrument” (BFAI) developed by Peters et al. [32, 33] was used, from which items were adapted to our study context. The survey (Online Resource 1) provided detailed information regarding the toolkit design with illustrative videos and figures, in English. The survey contained:

- Part I: general questions and statements on barriers and facilitators (BFAI statements)
- Part IIa: detailed questions on child maltreatment recognition
- Part IIb: open-ended questions regarding future child maltreatment toolkit implementation

BFAI statements were divided into statements concerning implementation of the comprehensive toolkit design and its individual parts (SCAN, training, and hospital policy). Answers on statements were based on a five-point Likert scale, ranging from 1 (fully disagree) to 5 (fully agree).

At the start of the survey, respondents needed to complete a mandatory question for informed consent. The pre-final version of the survey was pilot tested by different healthcare professionals and their comments were used to finalise the survey.

### Distribution survey

The survey was programmed in Limesurvey [34] and an open hyperlink was used for respondents to access the survey from September 2021 to August 2022, with the option to respond anonymously. To reach all healthcare professionals working in European EDs (stakeholders including doctors, nurses, trainees, managers, etc.), three methods for distribution were employed:

- Method 1: Key member approach
  o The REPEM network (Research in European Paediatric Emergency Medicine, pediatricians) was sent an email requesting to forward the introduction email and survey to 1–5 or 5–10 general hospitals in their country (depending on country size addressing one responsible contact person per hospital leading to 55 to 135 possible participants).
- Method 2: general approach
  o The EuSEN executive board asked EuSEN members (European Society for Emergency Nursing, nurses) to complete the survey.
  o At the EUSEM (European Society for Emergency Medicine) congress in 2021, at the Emergency Medicine day of EUSEM, and through EUSEM’s social media, visitors were asked through presentation slides, websites, and flyers to complete the survey. Researchers and ED professionals from all over Europe had access to these channels.

These two methods were also used for the distribution of the previous survey [27].

- Method 3: Previous respondents and additional contacts
  o Respondents from the previous survey [27] who filled in their contact details for follow-up questions (*n* = 96) were contacted (ED professionals).
  o In addition, contacts from different European networks (217 to 242 possible participants) were asked to complete the survey, including the MOFICHE (Management and Outcome of Fever in Children in Europe) network, IRG-MTS network (International Research Group Manchester Triage System), and Dutch PEM network (Pediatric Emergency Medicine), including mostly ED professionals and pediatricians.

### Statistical analysis

Respondents from non-European countries were excluded. The response rate was calculated based on the minimum and maximum number of respondents in conjunction with the responses from the REPEM (*n* = 55–135), previous respondents (*n* = 96), and additional contacts (*n* = 217–242). This yielded between *n* = 368 and *n* = 473 potential participants depending on the distribution of the survey through different REPEM key members (method 1) and some additional contacts (method 3), each tasked with contacting between 1 and 10 hospitals based on country size. Since the distribution through EUSEM and EuSEN (method 2) mainly took place via social media, no response rate could be calculated.

Duplicate responses (*n* = 5) were identified by concurrent personal contact details (i.e. name, e-mail and hospital) and merged to one response by taking the average from every answer.

Data were analysed using IBM SPSS Statistics 28. Descriptive analyses were performed regarding baseline characteristics and additional answers on child maltreatment recognition at the respondents’ hospitals.

The 5-point Likert scale answers were categorized into three groups: agree/fully agree (4 and 5 on Likert scale), inconclusive (3 on Likert scale) and disagree/fully disagree (1 and 2 on Likert scale). After categorizing statement answers into these groups, percentages were calculated based on the total number of participants who answered each statement, excluding missing responses (complete case analysis). Barriers and facilitators were identified in accordance with earlier studies [35–37]. Positively worded statements to which ≥ 20% of the healthcare professionals responded ‘(totally) disagree’ were considered barriers, and to which ≥ 80% responded ‘(totally) agree’ were considered facilitators. For negatively worded statements, if ≥ 20% (totally) agreed, statements were considered barriers and if ≥ 80% of participants (totally) disagreed, statements were considered facilitators. Additional Mann–Whitney U tests were used to test for statistical differences in barriers and facilitators between subgroups of respondents. A Bonferroni correction was applied to account for multiple comparisons, resulting in a significance threshold of *p* = 0.002. We analysed general versus university/teaching hospitals, mixed versus pediatric EDs and compared the group that stated “they already work according to the mentioned strategy or complete toolkit” (answered with ‘(fully) agree’) to the group that did not. Also, responses from female versus male and physician versus nurse were compared. These subgroups were selected to assess potential selection bias and the results’ generalizability.

For responses on open-ended questions a thematic analysis was performed, drawing on Braun and Clarke’s methodology to identify themes within qualitative data [38]. Recurrent terminology in these responses were identified and they were matched with commonly used themes in implementation research such as healthcare professional related and organizational levels [39].

## Results

### General information respondents

A total of 206 healthcare professionals from the different emergency societies and social media (*n* = 60), previous respondents (*n* = 53), and additional contacts (*n* = 93) participated. The estimated response rate ranged from 36% (*n* = 170/473) to 46% (*n* = 170/368). Non-European responses were excluded (*n* = 2). The remaining 204 responses from approximately 121 different hospitals in 28 European countries were analysed (Online Resource 2).

Of the respondents, 67.6% (*n* = 138/204) were (pediatric and/or emergency) physicians and 21.6% (*n* = 44/204) were nurses (Table 1). The majority of respondents worked at pediatric EDs (*n* = 98/204) or mixed EDs (*n* = 98/204), and 59% (*n* = 120/204) of respondents were female (Table 1).

**Table 1:** Baseline characteristics of respondents

Baseline characteristics respondents	Total *n* = 204 (%)
Profession stakeholders	
Paediatrician	54 (26.4)
Paediatric emergency physician	58 (28.4)
Emergency physician	26 (12.7)
ED nurse/(paediatric) nurse	44 (21.6)
ED manager	8 (3.9)
Resident/trainee emergency medicine	7 (3.4)
Other^	5 (2.5)
Type of hospital*	
General hospital	54
Teaching hospital	56
Academic/University hospital	118
Patients at your ED are	
Only adults	7 (3.4)
Both children and adults	98 (48.0)
Only children	98 (48.0)
Gender	
Female	120 (58.8)
Male	79 (38.7)
Unknown or missing	5 (2.5)

^*^Multiple answers possible; ^i.e. pediatric surgeon, social worker, etc.

### Facilitators for implementation

Regarding the toolkit design, 83.0% stated to be ‘willing to use it in the future’. In addition, facilitators were ‘having no problems with changing old routines’ (81.1%) and ‘the toolkit leaving enough room to make own conclusions’ (80.2%) (Table 2). The majority thought doctors/assistants (77.4%) and managers/directors (61.3%) would cooperate in applying this toolkit; however, these did not meet the definition for a facilitator (≥ 80%) (Table 2).

**Table 2:**
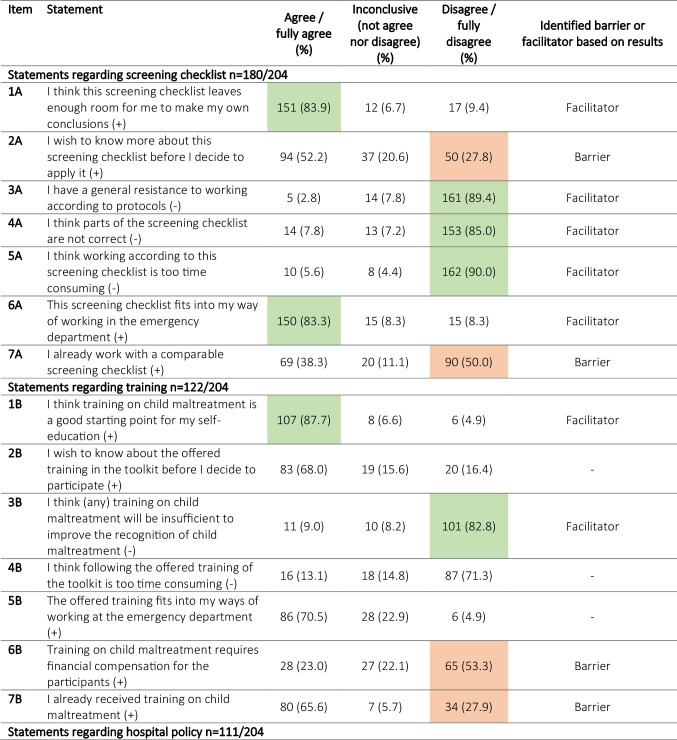

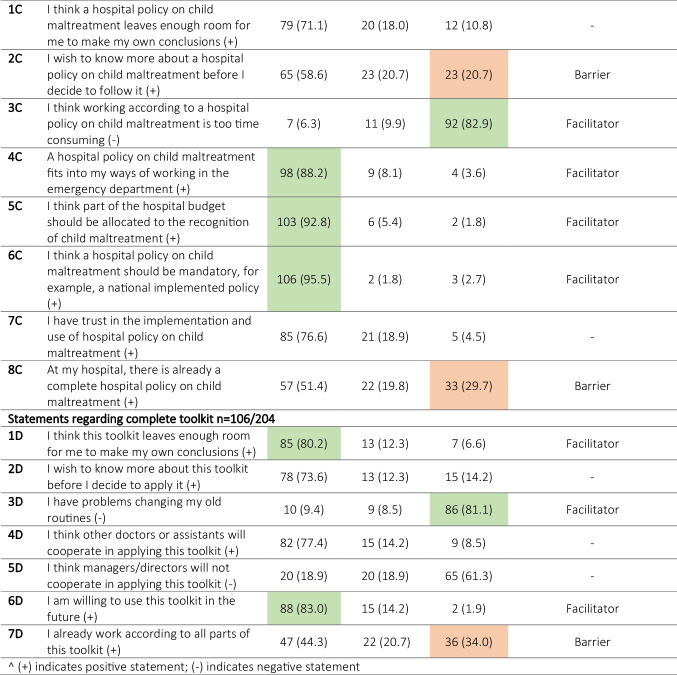
Barriers and facilitators defined based on statements on 5- point Likert scale on screening checklist, training, hospital policy and the comprehensive toolkit design including all 3 strategies

^ (+) indicates positive statement; (-) indicates negative statement

Facilitators are items to which ≥80% of health professionals responded with ‘totally disagree/disagree’ on negative statements and with ‘totally agree/agree’ on positive statements (highlighted in green). Barriers are items to which ≥20% of health professionals responded with ‘totally agree/agree’ on negative statements and with ‘totally disagree/disagree’ on positive statements (highlighted in orange).

For the individual parts of the toolkit, multiple facilitators were identified in accordance with those for the toolkit design (Table 2).

Facilitators for implementing the screening checklist were that it ‘leaves enough room for own conclusions’ (83.9%), ‘fits into way of working in the ED’ (83.3%), is ‘not too time-consuming’ (90.0%), is ‘not incorrect’ (85.0%) and ‘there is no general resistance amongst healthcare professionals to working according to protocols’ (89.4%).

‘Being a good starting point for self-education’ (87.7%) and ‘thought to be sufficient to improve recognition of child maltreatment’ (82.8%) were identified as facilitators for implementing the training.

Facilitators for implementing a hospital policy were comparable to those for the screening checklist: ‘fits into the way of working in the ED’ (88.2%) and is ‘not too time-consuming’ (82.9%). Other facilitators for implementation of a hospital policy were that it ‘should be mandatory (nationally implemented)’ (95.5%) and ‘part of the hospital budget should be allocated to child maltreatment recognition’ (92.8%). Most respondents stated to ‘have trust in the implementation and use of a hospital policy on child maltreatment’ (76.6%); however, this did not meet the definition of a facilitator.

### Barriers for implementation

The main barrier defined for all parts of the toolkit was the ‘wish to know more before deciding to apply them in practice’ (Table 2). The necessity for budget allocation corresponds to one other barrier, namely ‘requiring financial compensation for participants in the training’ (53.3%) (Table 2).

### Subgroup analysis of respondents and facilitators and barriers

Comparing responses from general hospitals (*n* = 46) to those from university/teaching hospitals (*n* = 158), and comparing responses from pediatric EDs (*n* = 98) to those from mixed EDs (*n* = 98) showed comparable facilitators and barriers for the toolkit design and its individual parts (Online Resource 3).

Respondents already using a comparable screening checklist more often identified ‘the screening checklist fitting into the way of working in the ED' as a facilitator (*n* = 63/69, 91.3%) compared with those not using such a strategy (*n* = 87/110, 79.1%; *p* < 0.001).

Comparing responses from physicians (*n* = 137) and nurses (*n* = 38) showed no significant differences in barriers and facilitators. No significant differences were found between responses from females (*n* = 120) and males (*n* = 79).

### Thematic analysis based on open-ended questions

A total of 53 participants responded to open-ended questions in part II of the survey. In general, the main reason for applying the toolkit design was to create awareness regarding child maltreatment and child safety. The main reason for not applying the toolkit was a lack of time.

**Fig. 1:**
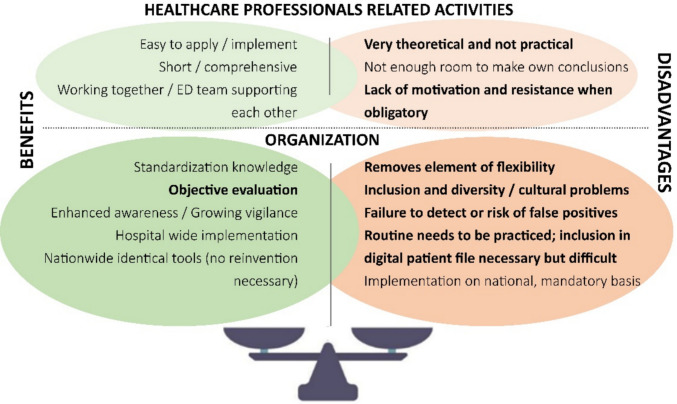
Thematic analysis from open-ended questions regarding the implementation of the child maltreatment toolkit, illustrating identified benefits and disadvantages by thematic analysis of open-ended questions. Bold benefits and disadvantages are new compared to the facilitators and barriers identified based on the survey statements, while neutral benefits and disadvantages are comparable

With thematic analysis, two main themes were identified: ‘Healthcare professionals related activities’ and ‘Organization’ (Fig. 1 and Table 3) [39]. Benefits of the toolkit within both themes were comparable with the mentioned facilitators in the BFAI statements (Fig. 1). A potential benefit stated by respondents, which was not identified with the BFAI statements, was ‘objective evaluation’ (Fig. 1).

**Table 3:** Responses to open-ended questions with thematic analysis

Question	Theme	Summary of comments of respondents
Which of the three parts of the toolkit do you think will cause the most problems?		
Checklist	Healthcare professionals related activities	- It can be clicked away/ignored - Poor adherence to checklists/policies - Should be short, fast and accurate, to reach full compliance
	Organization	- Possible false positives if applied to all children - Time-consuming/It needs time - Need for more clinical/case examples (practical and not theoretical learning)
Training	Healthcare professionals related activities	- All doctors will not participate/only staff of child protection group will be willing to take part on the training - People are resistant/problem with motivation/difficult to train those who are not interested - If non-committal, then many people won’t do it
	Organization	- Needs to be repeated certain times/repeated training efforts are required/re-training - Organization - Time-consuming/enough time to attend - Financial compensation/costs - English language
Hospital policy	Healthcare professionals related activities	- Hard to deviate from an old routine/lack of knowledge in hospital staff/some professionals don’t want to see and manage situations of child maltreatment
	Organization	- Lack of national requirements/whole region should have similar policy - Management/changes comes from above/it needs agreement from many professionals - Hospital wide implementation/hurdle to introduce new policies/involve all staff - Budget/money - Very time-consuming and slow process - Language
What do you think is the greatest benefit of this toolkit?	Healthcare professionals related activities	- Easy to handle, quick and efficient/comprehensive, clear and easy to apply/easy to use/short and accurate/shorter checklist - Rather easy to implement into the every day routines of the ED - ED team supporting each other/working together in the chain of care
	Organization	- Standardized way to improve early detection and recognition of child maltreatment/better sensitivity/Accuracy and objective evaluation/Enhanced awareness/growing vigilance/standardization knowledge - Hospital wide implementation - Identical tools nationwide, no need for each hospital to reinvent
What do you think is the greatest disadvantage of a toolkit for the recognition of child maltreatment?	Healthcare professionals related activities	- Lack of motivation/danger of ignorance/making it compulsory will get negative feedback/resistance because it is difficult to switch to something else - Very theoretical and not practical - Stops people thinking for themselves/not using their own brain/not enough room to make own conclusions
	Organization	- Failure to detect significant portion of cases/do not know sensitivity/risk of false positives/even with normal toolkit children could be maltreated/overestimation/if not validated can miss children at risk - Implementation necessary on a national, mandatory basis/(standardized) implementation - Should be reflected in the ED chart but difficult to get it included in electronic chart - Inclusion and diversity/cultural problems - Removes element of flexibility - Routine needs to be practiced - Time-consuming/lack of time
Could you name one or more circumstances or situations that would make it easier for you to apply this toolkit?	Healthcare related activities	- Communication - Better argumentation with parents - Interactive exercises/examples of practical and clinical cases/educational material and regular training
	Organization	- Digital application in electronic patient record/documentation for extra information - Implementation nationwide/national requirements and law - Fixed part of the admission/mandatory for everybody/mandatory training/standard and must do - Shortness of time - Triage zone of busy ED/when ED is busy - Money for implementation and training
Could you name one or more circumstances or situations that would make it harder for you to apply this toolkit?	Healthcare professionals related activities	- High work flow/acute life-threatening situations/busy triage zone/rush hours/stress due to increased patient volume/high case load/daily overcrowding in the ED - Lack of motivation - Difficulties finding it or filling it in/ongoing challenges with documentation - Parents oppositional - When children are managed exclusively by traumatologist or surgeons
	Organization	- ICT failure if not incorporated into downtime forms - No prompting or support/only implement through official directive - Time constraints - Staff shortages - It is not paid - Linguistic barrier
What would be the main reason for you to apply this toolkit?	Healthcare professionals related activities	- Easy to use/easy/short and clear - Awareness/augmented patient safety/better protection of children/child safety/efficacy - Better argumentation with parents
	Organization	- Hospital wide introduction/standardized guidelines - Mandated and incorporated in digital patient file as a prompt - Mandatory obligation to report/act according to law requirements - Having time/does not waste a lot of time
What would be the main reason for you to not apply this toolkit	Healthcare professionals related activities	- Fear of false accusations/false positives
	Organization	- Not used by everyone/not agreed by Hospital Violence Commission/lack of acknowledgement and feedback - No general obligation - Too expensive/no funding for training/no money/not being able to afford it - Lack of time/time-consuming

For both themes, potential disadvantages not mentioned in BFAI statements were: ‘very theoretical and not practical’, ‘lack of motivation and resistance when obligatory’, ‘removes element of flexibility’, ‘cultural problems issues’, ‘integrating inclusion in digital patient file’ and ‘fear of risk of false positives’ (Fig. 1). The details of the thematic analysis are described in Table 3.

## Discussion

This pre-implementation survey amongst healthcare professionals at European EDs showed that the presented child maltreatment toolkit design consisting of the SCAN screening checklist, training and hospital policy, was positively received. The main perceived reason for the toolkit’s application was improving vigilance and structured recognition of child maltreatment and the main perceived benefit was hospital-wide (and national) implementation. A diversity of facilitators was identified for all individual parts of the toolkit based on the BFAI statements, generally relating to the toolkit fitting into the ED’s dynamic working environment. Key facilitators for all parts of the toolkit consisted of time, financial and staff resources, motivation of ED staff and specifically for the hospital policy standardized hospital-wide implementation, moreover mandatory and national implementation. For training, the necessity of financial compensation for participants was identified as an important barrier. In addition, thematic analysis showed objective evaluation as an important benefit of the toolkit design. Potential disadvantages were cultural differences, risk of false positives, challenges with integration into electronic health records and guaranteeing practice over theory.

Previous studies regarding facilitators of and barriers to child maltreatment screening at the ED showed similar results, but did not assess screening combined with training and hospital policy implementation. Louwers et al. [23] identified resource constraints and time as important barriers for both ED staff and board members in screening for child abuse and improving policies. The study underscores the need for a comprehensive protocol in EDs, including a screening instrument and follow-up procedures [23].

The World Health Organisation (WHO) guidelines advise against universal screening due to the risk of false-positive child abuse cases and other potential harms, equity considerations, system capacity limitations and the need for ongoing evaluation [40]. Importantly, this study does not evaluate the effectiveness of universal screening nor advocate unqualified implementation across Europe. Rather, it identifies perceived determinants that must be addressed before any safe evaluation or adoption. The WHO 2020 screening criteria emphasize demonstrated benefit, minimized harm, system integration and data-driven quality improvement [41]. The proposed toolkit design does not in itself fulfil these criteria but incorporates elements such as structured follow-up, governance and training, that may support alignment if prospectively evaluated.

The SCAN [10] is intended to identify children potentially at increased risk, not to establish a diagnosis. Given its reported sensitivity and positive predictive value [10], careful contextual interpretation is essential. A positive screening result should therefore trigger a structured secondary assessment pathway, including comprehensive history-taking, full physical examination, multidisciplinary consultation, and clearly defined documentation and decision thresholds embedded within hospital policy. Such a pathway is particularly crucial in jurisdictions with mandatory reporting [42], where a positive screen must not automatically equate to referral but should be embedded within defined expert review and safeguarding procedures.

Overcrowding at the ED was one of the main concerns of respondents regarding successful implementation and use of the child maltreatment toolkit. As a solution, one of the main expected benefits of the toolkit could be standardized implementation, if possible mandatory and nationally. Structured processes and protocols have been associated with reduced clinical error rates [43], which is particularly relevant in complex and sensitive situations such as suspected child maltreatment. Comparable barriers and facilitators were reported across paediatric and mixed EDs, suggesting that contextual adaptation rather than setting type may be the primary consideration for future implementation. Given that the ED is often the first point of contact for vulnerable children, establishing reliable and context-sensitive procedures may support timely recognition, appropriate safeguarding actions and coordination within the broader child protection system [8].

Concerns about false positives and missed cases were explicitly raised by respondents. These concerns reinforce the need for prospective monitoring of detection rates, referral patterns, equity indicators, and unintended consequences. Training remains essential not only to support correct use of SCAN but also to mitigate interpretive bias and enhance communication skills. Real-time case discussion and practical supervision were identified as important facilitators [24].

Cultural and legal variability across Europe further necessitates contextual adaptation. Implementation should explicitly consider local reporting laws, system capacity, and documentation standards.

This study focuses on healthcare professionals’ perceptions of implementation determinants rather than clinical effectiveness. Its strength lies in providing a cross-European overview of perceived barriers and facilitators as a foundation for further co-design, refinement and prospective evaluation. Limitations are that responses from individual professionals do not reflect the opinion of all hospital staff, highlighting a recognized limitation in survey designs [44]. Nonetheless, there were no major differences in barriers and facilitators between subgroups of respondents. University/teaching hospitals are often larger with more specialised teams and protocols compared to general hospitals which have fewer resources. While this might have affected the results, subgroup analyses showed comparable barriers and facilitators. The response rate was acceptable according to the literature [45] but a selection bias could have occurred with possible overrepresentation of university/teaching hospitals and hampering non-member healthcare professionals by contacting mostly existing emergency medicine networks.

In conclusion, while the toolkit design was positively received, its broader adoption requires careful contextualization, structured governance, and prospective hybrid effectiveness–implementation evaluation to assess both benefits and potential harms. Addressing identified barriers, particularly resource allocation, legal variability, and concerns about screening performance, will be essential before wider implementation.

## Supplementary Information

Below is the link to the electronic supplementary material.

ESM 1 Supplementary Material 1 (DOCX 11.1 MB)

## Data Availability

All relevant data are within the manuscript and its Supporting Information files. The data underlying the results presented in the study are available upon reasonable request.

## Abbreviations

BFAI: Barriers and Facilitators Assessment Instrument
ED: Emergency Department
EHR: Electronic Health Record
EUSEM: European Society for Emergency Medicine
EuSEN: European Society for Emergency Nursing
IRG-MTS: International Research Group Manchester Triage System
MOFICHE: Management and Outcome of Fever in Children in Europe.
NICE: National Institute for Health and Care Excellence
PEM: Pediatric Emergency Medicine
REPEM: Research in European Paediatric Emergency Medicine
SCAN: Screening Instrument for Child Abuse & Neglect
WHO: World Health Organisation
