# Supplementary material for: Improving the recognition of child maltreatment in emergency departments in Europe: healthcare professionals’ perceived barriers and facilitators for implementation of a comprehensive toolkit design
Source: Eur J Pediatr. 2026 Apr 8;185(5):242. doi: 10.1007/s00431-026-06815-8 (PMC13061767; doi:10.1007/s00431-026-06815-8)
Supplement: Supplementary file 1 — Supplementary Material 1 (DOCX 11.1 MB) [file 431_2026_6815_MOESM1_ESM.docx]

**Supplementary files *‘Improving the recognition of child maltreatment in emergency departments in Europe: barriers and facilitators for implementation of a complete toolkit’***

**Authors**: F. Hoedeman^1^, P.J. Puiman^1^, A.W. Smits^2^, M.I. Dekker^2^, D. Lauwaert^3***^, R. Oostenbrink^1**^, N. Parri^4*^, L. García-Castrillo Riesgo^5*^, S. Polinder^6^, H.A. Moll^1^

**On behalf of the European Society for Emergency Medicine (EUSEM) research committee, **Research in European Pediatric Emergency Medicine (REPEM) and ***European Society for Emergency Nursing (EuSEN)*

**Affiliations:**

1. Department of General Paediatrics, Erasmus MC-Sophia Children’s Hospital, Rotterdam, The Netherlands

2. Augeo Foundation, Driebergen, The Netherlands

3. Emergency Department, University Hospital Brussels, Brussels, Belgium

4. Meyers Children’s Hospital IRCCS, Florence, Italy

5. Emergency Department, University Hospital Marques Valdecilla, Cantabria, Spain

6. Department of Public Health, Erasmus University Medical Centre, Rotterdam, The Netherlands

**Corresponding author:** Dr. Patrycja J. Puiman, [p.puiman@erasmusmc.nl](mailto:p.puiman@erasmusmc.nl)

**Content supplementary file**

**Online Resource 1:** Survey on barriers and facilitators of a child maltreatment toolkit

**Online Resource 2:** Number of responses per European country

**Online Resource 3:** Subgroup analysis barriers and facilitators general versus teaching/university hospitals and mixed versus pediatric EDs

**Online Resource 1: Survey on barriers and facilitators of a child maltreatment toolkit**

**Survey title: Complete toolkit for recognition of child maltreatment**

***Consent***

**By proceeding with this questionnaire, you are consenting to participate in this study.**

- Proceed

***Part I General information and barriers & facilitators questions of the toolkit***

1. **What country do you work in?**

- Belgium
- Croatia
- Cyprus
- Denmark
- Estonia
- Finland
- France
- Georgia
- Germany
- Greece
- Hungary
- Iceland
- Ireland
- Italy
- Latvia
- Lithuania
- Malta
- Netherlands
- Norway
- Poland
- Portugal
- Serbia
- Slovakia
- Slovenia
- Spain
- Sweden
- Switzerland
- Turkey
- United Kingdom
- Other, specify….

1. **What is your gender?**

- Male
- Female

1. **What is your profession?**

- Paediatrician
- Paediatric emergency physician
- Emergency physician
- ED nurse
- Nurse
- ED manager
- Resident / trainee
- Other, specify…….

1. **Where do you work? Please state the name and location (city) of your hospital:**
2. **In what type of hospital do you work?**

- General/rural hospital
- Teaching/Academic/University hospital
- Other, specify………

1. **Patients at your ED are:**

- Only adults
- Both children and adults
- Only children

**Implementation of a toolkit for the recognition of child maltreatment**

To improve the detection and recognition of child maltreatment at the emergency department we offer a toolkit, consisting of:

1. *Screening checklist*: a validated screening checklist (the SCAN[1]) including items which trigger further evaluation: injury fits with developmental level and history, delay in seeking medical help, appropriate behaviour/interaction child and carers, and signals that make you doubt about the safety of child/family.
2. *Training*: training with focus on the recognition of signs and symptoms in children (child characteristics) and also on the recognition of parental characteristics or risk factors in parents admitted to the emergency department such as patients with severe psychiatric problems, substance abuse and domestic violence.
3. *Hospital policy:* a written policy describing necessary items including: availability of a checklist, training, a local child abuse team and dedicated professionals on child maltreatment to guide appropriate actions.

1] Hoedeman F, Puiman PJ, van den Heuvel EAL, et al. A validated Screening instrument for Child Abuse and Neglect (SCAN) at the emergency department. Eur J Pediatr. 2023 Jan;182(1):79-87. doi: 10.1007/s00431-022-04635-0.

1] Hoedeman F, Puiman PJ, van den Heuvel EAL, et al. A validated Screening instrument for Child Abuse and Neglect (SCAN) at the emergency department. Eur J Pediatr. 2023 Jan;182(1):79-87. doi: 10.1007/s00431-022-04635-0.

**Screening checklist** for the recognition of child maltreatment

Systematic screening can improve the detection rate of suspected child abuse. The Screening instrument for Child Abuse & Neglect (SCAN) is a validated checklist for child maltreatment (child characteristics): injury fits with developmental age and history, delay in seeking medical help, behaviour/interaction between child and carers, and doubting of professionals about safety.[1] The checklist needs to be completed for all children visiting the emergency department and only takes a couple of minutes to complete.

Screenshot screening checklist[1]:

*

*

*There follows a number of statements about the implementation of the screening checklist. We would like to know whether you agree with each statement or not and to what degree. If you don’t have a strong opinion, please try to decide if it is more like ‘agree’ or more like ‘disagree’. If you really don’t know, you can select the option ‘do not agree nor disagree’.*

| **Statements screening checklist** | Fully  Disagree | Disagree | Do not agree nor disagree | Agree | Fully Agree |
| --- | --- | --- | --- | --- | --- |
| 1. I think this screening checklist leaves enough room for me to make my own conclusions |  |  |  |  |  |
| 2. I wish to know more about this screening checklist before I decide to apply it |  |  |  |  |  |
| 3. I have a general resistance to working according to protocols |  |  |  |  |  |
| 4. I think parts of this screening checklist are not correct |  |  |  |  |  |
| 5. I think working according to this screening checklist is too time consuming |  |  |  |  |  |
| 6. This screening checklist fits into my way of working in the Emergency Department |  |  |  |  |  |
| 7. I already work with a comparable screening checklist |  |  |  |  |  |

| **Training** regarding the recognition of child maltreatment  Professionals trained in the detection of child abuse are more likely to recognize child maltreatment and take appropriate action if they have concerns about a particular patient. Training will not only focus on the recognition of child maltreatment based on child characteristics (signs and symptoms) but also on the recognition of child maltreatment based on parental risk factors such as psychiatric problems, substance abuse or domestic violence.  The training currently offered in the toolkit consists of an e-learning programme which will take 3 hours to complete in total. The e-learning consists of two interactive modules with video-examples and exercises: one module (2 hours) on the recognition of signs of child maltreatment (based on signs which you can see in children or in parents) and how to act upon these signs, and one module on communication about violence (1 hour). The modules can be paused and re-played at any time.  See demo child characteristics: [https://www.augeo.nl/Demo/Demo%20Residency%20training/](https://eur01.safelinks.protection.outlook.com/?url=https%3A%2F%2Fwww.augeo.nl%2FDemo%2FDemo%2520Residency%2520training%2F&data=04%7C01%7Cf.hoedeman%40erasmusmc.nl%7Cdc19288b65794bf180b808d904aa593a%7C526638ba6af34b0fa532a1a511f4ac80%7C0%7C0%7C637545951958244440%7CUnknown%7CTWFpbGZsb3d8eyJWIjoiMC4wLjAwMDAiLCJQIjoiV2luMzIiLCJBTiI6Ik1haWwiLCJXVCI6Mn0%3D%7C1000&sdata=IEWsBo%2F4KqXQAJUwI3VHNcROCh%2BEBzgy6KRTWeca1%2Bo%3D&reserved=0)  See demo communication: [https://www.augeo.nl/Demo/140520_PROMO-Communiceren%20over%20Geweld-v2014-ENG_mvi.mp4](https://eur01.safelinks.protection.outlook.com/?url=https%3A%2F%2Fwww.augeo.nl%2FDemo%2F140520_PROMO-Communiceren%2520over%2520Geweld-v2014-ENG_mvi.mp4&data=04%7C01%7Cf.hoedeman%40erasmusmc.nl%7Cdc19288b65794bf180b808d904aa593a%7C526638ba6af34b0fa532a1a511f4ac80%7C0%7C0%7C637545951958254431%7CUnknown%7CTWFpbGZsb3d8eyJWIjoiMC4wLjAwMDAiLCJQIjoiV2luMzIiLCJBTiI6Ik1haWwiLCJXVCI6Mn0%3D%7C1000&sdata=BiVRIukOcMa2OaNv11u87Tq3PQw%2F%2F4%2FRMbjqFLhDExc%3D&reserved=0)  *There follows a number of statements about the implementation of the training. We would like to know whether you agree with the statement or not and to what degree. If you don’t have a strong opinion, please try to decide if it is more like ‘agree’ or more like ‘disagree’. If you really don’t know, you can select the option ‘do not agree nor disagree’.* | | | | | |
| --- | --- | --- | --- | --- | --- |
| **Statements training on child maltreatment** | Fully  Disagree | Disagree | Do not agree nor disagree | Agree | Fully Agree |
| 1. I think training on child maltreatment is a good starting point for my self-education |  |  |  |  |  |
| 2. I wish to know more about the offered training in the toolkit before I decide to participate |  |  |  |  |  |
| 3. I think (any) training on child maltreatment will be insufficient to improve the recognition of child maltreatment |  |  |  |  |  |
| 4. I think following the offered training of the toolkit is too time consuming |  |  |  |  |  |
| 5. The offered training fits into my ways of working at the Emergency Department |  |  |  |  |  |
| 6. Training on child maltreatment requires financial compensation for the participants |  |  |  |  |  |
| 7. I already received training on child maltreatment |  |  |  |  |  |

| **Hospital policy protocol** regarding the recognition of child maltreatment  A hospital policy on child maltreatment is recorded in a written protocol describing standard care including assessment and management of patients with suspected child maltreatment. This protocol sets the foundation within the hospital for the delivery of safe and cost-effective quality care. The organisation of hospital facilities is documented in the protocol, such as the presence of a child abuse team and/or child abuse policy officer to help facilitate the recognition of child maltreatment and for monitoring or referring. Also, providing education, a (digital) screening checklist and follow-up guidelines on how to act in case of a positive screening result are recorded in such a protocol. Other items described in the protocol are guidelines on communication with child protective services and/or child abuse centers and the presence of a financial budget for child maltreatment.  *There follows a number of statements about working according to the hospital policy regarding the recognition of child maltreatment.. We would like to know whether you agree with the statement or not and to what degree. If you don’t have a strong opinion, please try to decide if it is more like ‘agree’ or more like ‘disagree’. If you really don’t know, you can select the option ‘do not agree nor disagree’.* | | | | | |
| --- | --- | --- | --- | --- | --- |
| **Statements** **hospital** **policy** | Fully  Disagree | Disagree | Do not agree nor disagree | Agree | Fully Agree |
| 1. I think a hospital policy on child maltreatment leaves enough room for me to make my own conclusions |  |  |  |  |  |
| 2. I wish to know more about a hospital policy on child maltreatment before I decide to follow it |  |  |  |  |  |
| 3. I think working according to a hospital policy on child maltreatment is too time consuming |  |  |  |  |  |
| 4. A hospital policy on child maltreatment fits into my ways of working in the Emergency Department |  |  |  |  |  |
| 5. I think a part of the hospital budget should be allocated to the recognition of child maltreatment |  |  |  |  |  |
| 6. I think a hospital policy on child maltreatment should be mandatory, for example, a national implemented policy |  |  |  |  |  |
| 7. I have trust in the implementation and use of a hospital policy on child maltreatment |  |  |  |  |  |
| 8. At my hospital, there is already a complete hospital policy on child maltreatment |  |  |  |  |  |

**Implementation of a toolkit for the recognition of child maltreatment**

To improve the detection and recognition of child maltreatment in the emergency department we would like to offer a toolkit, consisting of:

1. *Screening checklist*: a validated screening checklist (the SCAN[1]) including items such as injury fits with developmental level and history, delay in seeking medical help, appropriate behaviour/interaction child and carers, and signals that make you doubt about the safety of child/family. A positive screening item prompts further evaluation.
2. *Training*: training will not only focus on the recognition of child characteristics but also on risk factors in parents admitted to the emergency department such as severe psychiatric problems, substance abuse and domestic violence.
3. *Hospital policy*: a hospital policy for a screening checklist and training to be available and for a team/dedicated professional to guide appropriate actions.

*There follows a number of statements about the implementation of such a toolkit. We would like to know whether you agree with the statement or not and to what degree. If you don’t have a strong opinion, please try to decide if it is more like ‘agree’ or more like ‘disagree’. If you really don’t know, you can select the option ‘do not agree nor disagree’.*

| **Statements toolkit** | Fully  Disagree | Disagree | Do not agree nor disagree | Agree | Fully Agree |
| --- | --- | --- | --- | --- | --- |
| 1. I think this toolkit leaves enough room for me to make my own conclusions |  |  |  |  |  |
| 2. I wish to know more about this toolkit before I decide to apply it |  |  |  |  |  |
| 3. I have problems changing my old routines |  |  |  |  |  |
| 4. I think other doctors or assistants will cooperate in applying this toolkit |  |  |  |  |  |
| 5. I think managers/directors will cooperate in applying this toolkit |  |  |  |  |  |
| 6. I am willing to use this toolkit in the future |  |  |  |  |  |
| 7. I already work according to all parts of this toolkit |  |  |  |  |  |

1] Hoedeman F, Puiman PJ, van den Heuvel EAL, et al. A validated Screening instrument for Child Abuse and Neglect (SCAN) at the emergency department. Eur J Pediatr. 2023 Jan;182(1):79-87. doi: 10.1007/s00431-022-04635-0.

***End of Part I***

You have reached the end of Part I of the survey. Thank you for answering these questions. To obtain more information on the current situation regarding child maltreatment at your hospital and on the toolkit, we would appreciate it if you could complete some additional questions in Part II.

- Continue to Part II
- Continue to end of the survey

***Part II Additional questions on recognition of child maltreatment at your hospital:***

1. **Does your hospital have a standardised policy or guideline for the detection of child abuse?**

- Yes
- No
- Other, please specify…………………………………………………………………
- Don’t know

1. **Is there a child abuse/child protection team and/or a child abuse & neglect policy officer in your hospital?**

- Yes
- No
- Other, please specify…………..……………………………………………………
- Don’t know

**3A. Do you use a screening tool or checklist to detect suspicion of child abuse?**

- No screening checklist is used
- Yes

**3B. When is the screening tool or checklist used?**

- For all children who enter the ED
- When there is a suspicion of child abuse
- For certain risk groups, please specify……………………………………..

**4. Have you taken part in a training programme for detecting child abuse based on child characteristics over the last 3 years? (Multiple answers possible)**

- Yes, in-hospital training
- Yes, regional training
- Yes, national training
- Other, please specify…………………………………………………………
- No

**5. Do you have any guidelines in your hospital and/or have you taken part in any training programme for the detection of child maltreatment based on parental risk factors*?**

- Yes
- No
- Other, please specify.………… ……………………………………………………
- We don’t see adult patients
- Don’t know

******* *Children of parents who suffer from severe psychiatric problems, substance abuse or domestic violence, have a high risk of being or becoming victims of child maltreatment. The following questions refer to the procedure regarding an adult patient being a parent visiting the ED. In other words, when the parent is the patient, not the child.*

***Additional (open) questions on the toolkit:***

**6. Which of the three parts of the toolkit do you think will cause the most problems?**

- Checklist, please specify….
- Training, please specify…
- Hospital policy, please specify…
- None, please specify…..

**7. What do you think is the greatest benefit of this toolkit?**

….

**8. What do you think is the greatest disadvantage of this toolkit?**

....

**9. Could you name one or more circumstances or situations, that would make it easier for you to apply this toolkit?**

…..

**10. Could you name one or more circumstances or situations, that would make it harder for you to apply this toolkit?**

…..

**11. What would be the main reason for you to apply this toolkit?**

…..

**12. What would be the main reason for you not to apply this toolkit?**

.....

**You have reached the end of the survey**

**Do you have any questions or comments with regard to this survey?**

….

**We would like to contact you to inform you of the results and to ask any additional follow-up questions. Please leave your name and contact details (e-mail address). We will only use your personal data for the purposes described and in compliance with the General Data Protection Regulation.**

Name: ….

E-mail address: …

Thank you for completing the survey!

*
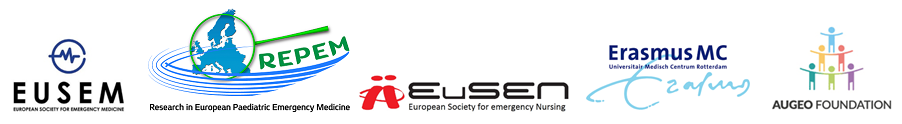
*

**Online Resource 2: Number of responses per European country**

| **Country** | **Number of respondents**  **(n=204)** |
| --- | --- |
|  |  |
| Austria^1^ | 20 |
| Belgium^2^ | 18 |
| Czech republic^1^ | 3 |
| Estonia^1^ | 2 |
| France^1^ | 6 |
| Germany^2^ | 10 |
| Greece^3^ | 1 |
| Hungary^1^ | 7 |
| Iceland^1^ | 1 |
| Ireland^2^ | 2 |
| Italy^1^ | 8 |
| Latvia^3^ | 1 |
| Liechtenstein^3^ | 1 |
| Lithuania^1^ | 1 |
| Malta^2^ | 2 |
| Montenegro^3^ | 1 |
| Netherlands^2^ | 30 |
| Norway^1^ | 2 |
| Poland^3^ | 2 |
| Portugal^3^ | 2 |
| Romania^3^ | 2 |
| Slovakia^3^ | 3 |
| Slovenia^1^ | 2 |
| Spain^1^ | 25 |
| Sweden^1^ | 23 |
| Switzerland^1^ | 7 |
| Turkey^3^ | 10 |
| United Kingdom^2^ | 12 |

Overview of number of responses on the survey per country. Mandatory reporting information was based on Otterman et al.[47], and shown per country in superscript with 1 = mandatory reporting, 2= no mandatory reporting, 3 = unknown mandatory reporting.

**Online Resource 3: Subgroup analysis barriers and facilitators general versus teaching/university hospitals and mixed versus pediatric EDs**

| Item | Statement | General hospital (n=46) | | | Teaching/university hospital (n=158) | | | Mixed EDs (n=98) | | | Pediatric EDs (n=98) | | |  |
| --- | --- | --- | --- | --- | --- | --- | --- | --- | --- | --- | --- | --- | --- | --- |
|  |  | Agree / fully agree (%) | Inconclusive (not agree nor disagree) (%) | Disagree / fully disagree (%) | Agree / fully agree (%) | Inconclusive (not agree nor disagree) (%) | Disagree / fully disagree (%) | Agree / fully agree (%) | Inconclusive (not agree nor disagree) (%) | Disagree / fully disagree (%) | Agree / fully agree (%) | Inconclusive (not agree nor disagree) (%) | Disagree / fully disagree (%) |  |
| Statements regarding screening checklist n=180/204 | | **N=36/46** | | | **N=144/158** | | | **N=86/98** | | | **N=87/98** | | |  |
| 1A | I think this screening checklist leaves enough room for me to make my own conclusions (+) | **33 (91.7)** | **2 (5.6)** | **1 (2.8)** | **118 (81.9)** | **10 (6.9)** | **16 (11.1)** | 70 (81.4) | 6 (7.0) | 10 (11.6) | 75 (86.2) | 5 (5.7) | 7 (8.0) |  |
| 2A | I wish to know more about this screening checklist before I decide to apply it (+) | 18 (39.1) | 10 (27.8) | 8 (22.2) | 76 (52.8) | 27 (18.8) | 42 (29.2) | **40 (46.5)** | **19 (22.1)** | **28 (32.6)** | **54 (62.1)** | **16 (18.4)** | **17 (19.5)** |  |
| 3A | I have a general resistance to working according to protocols (-) | 1 (2.8) | 4 (11.1) | 31 (86.1) | 4 (2.8) | 10 (6.9) | 130 (90.3) | 1 (1.2) | 7 (8.2) | 78 (90.7) | 4 (4.6) | 7 (8.0) | 76 (87.4) |  |
| 4A | I think parts of the screening checklist are not correct (-) | 3 (8.3) | 4 (11.1) | 29 (80.6) | 11 (7.6) | 9 (6.3) | 124 (86.1) | **8 (9.3)** | **8 (9.3)** | **70 (81.4)** | **5 (5.7)** | **5 (5.7)** | **77 (88.5)** |  |
| 5A | I think working according to this screening checklist is too time consuming (-) | 3 (8.3) | 2 (5.6) | 31 (86.1) | 7 (4.9) | 6 (4.2) | 131 (91.0) | 2 (2.3) | 6 (7.0) | 78 (90.7) | 8 (9.2) | 2 (2.3) | 77 (88.5) |  |
| 6A | This screening checklist fits into my way of working in the emergency department (+) | 30 (83.3) | 5 (13.9) | 1 (2.8) | 120 (83.3) | 10 (6.9) | 14 (9.7) | 66 (76.7) | 12 (14.0) | 8 (9.3) | 77 (88.5) | 3 (3.4) | 7 (8.0) |  |
| 7A | I already work with a comparable screening checklist (+) | 11 (30.6) | 5 (13.9) | 20 (55.6) | 58 (40.3) | 15 (10.4) | 70 (48.6) | 36 (41.9) | 9 (10.5) | 40 (46.5) | 33 (37.9) | 10 (11.5) | 44 (50.6) |  |
| Statements regarding training n=122/204 | | **N=27/46** | | | **N=94/158** | | | **N=63/98** | | | **N=56/98** | | |  |
| 1B | I think training on child maltreatment is a good starting point for my self-education (+) | 24 (88.9) | 2 (7.4) | 1 (3.7) | 83 (88.3) | 6 (6.4) | 5 (5.3) | 55 (87.3) | 3 (4.8) | 4 (6.3) | 49 (87.5) | 5 (8.9) | 2 (3.6) |  |
| 2B | I wish to know about the offered training in the toolkit before I decide to participate (+) | 19 (70.3) | 6 (22.2) | 2 (7.4) | 64 (68.1) | 13 (13.8) | 18 (19.1) | 43 (68.3) | 10 (15.9) | 10 (15.9) | 39 (69.6) | 8 (14.3) | 9 (16.1) |  |
| 3B | I think (any) training on child maltreatment will be insufficient to improve the recognition of child maltreatment (-) | 2 (7.4) | 2 (7.4) | 23 (85.2) | 9 (9.6) | 8 (8.5) | 78 (83.0) | 4 (6.3) | 5 (7.9) | 54 (85.7) | 7 (12.5) | 5 (8.9) | 44 (78.6) |  |
| 4B | I think following the offered training of the toolkit is too time consuming (-) | 3 (11.1) | 1 (3.7) | 23 (85.2) | 13 (13.8) | 17 (18.1) | 64 (68.1) | 9 (14.3) | 8 (12.7) | 45 (71.4) | 6 (10.7) | 10 (17.9) | 40 (71.4) |  |
| 5B | The offered training fits into my ways of working at the emergency department (+) | 20 (74.1) | 6 (22.2) | 1 (3.7) | 66 (70.2) | 22 (23.4) | 5 (5.3) | 42 (66.7) | 17 (27.0) | 2 (3.1) | 42 (75.0) | 11 (19.6) | 3 (5.4) |  |
| 6B | Training on child maltreatment requires financial compensation for the participants (+) | **10 (37.0)** | **7 (25.9)** | **10 (37.0)** | **18 (19.1)** | **20 (21.3)** | **55 (58.5)** | 14 (22.2) | 15 (23.8) | 32 (50.8) | 13 (23.2) | 11 (19.6) | 32 (57.1) |  |
| 7B | I already received training on child maltreatment (+) | 17 (63.0) | 1 (3.7) | 9 (33.3) | 63 (67.0) | 6 (6.4) | 25 (26.6) | 43 (68.3) | 3 (4.8) | 16 (25.4) | 35 (62.5) | 4 (7.1) | 17 (30.4) |  |
| Statements regarding hospital policy n=111/204 | | **N=25/46** | | | **N=86/158** | | | **N=54/98** | | | **N=54/98** | | |  |
| 1C | I think a hospital policy on child maltreatment leaves enough room for me to make my own conclusions (+) | 15 (60.0) | 4 (16.0) | 6 (24.0) | 64 (74.4) | 16 (18.6) | 6 (7.0) | 39 (72.2) | 7 (13.0) | 8 (14.8) | 37 (68.5) | 13 (24.1) | 4 (7.4) |  |
| 2C | I wish to know more about a hospital policy on child maltreatment before I decide to follow it (+) | 12 (48.0) | 9 (36.0) | 4 (16.0) | 53 (6.2) | 14 (16.3) | 19 (22.1) | 26 (48.1) | 15 (27.8) | 13 (24.1) | 36 (66.7) | 8 (14.8) | 10 (18.5) |  |
| 3C | I think working according to a hospital policy on child maltreatment is too time consuming (-) | 1 (4.0) | 3 (12.0) | 20 (80.0) | 6 (7.0) | 8 (9.3) | 72 (83.7) | 0 (0.0) | 7 (13.0) | 46 (85.2) | 7 (13.0) | 4 (7.4) | 43 (79.6) |  |
| 4C | A hospital policy on child maltreatment fits into my ways of working in the emergency department (+) | 23 (92.0) | 1 (4.0) | 1 (4.0) | 75 (87.2) | 8 (9.3) | 3 (3.5) | 46 (85.2) | 6 (11.1) | 2 (3.7) | 49 (90.7) | 3 (5.6) | 2 (3.7) |  |
| 5C | I think part of the hospital budget should be allocated to the recognition of child maltreatment (+) | 23 (92.0) | 1 (4.0) | 1 (4.0) | 80 (93.0) | 5 (5.8) | 1 (1.2) | 48 (88.9) | 4 (7.4) | 2 (3.7) | 52 (96.3) | 2 (3.7) | 0 (0.0) |  |
| 6C | I think a hospital policy on child maltreatment should be mandatory, for example, a national implemented policy (+) | 24 (96.0) | 0 (0.0) | 1 (4.0) | 82 (95.3) | 2 (2.3) | 2 (2.3) | 52 (96.3) | 1 (1.9) | 1 (1.9) | 52 (96.3) | 1 (1.9) | 1 (1.9) |  |
| 7C | I have trust in the implementation and use of hospital policy on child maltreatment (+) | 22 (88.0) | 3 (12.0) | 0 (0.0) | 63 (73.3) | 18 (20.9) | 5 (5.8) | 40 (74.1) | 12 (22.2) | 2 (3.7) | 43 (79.6) | 9 (16.7) | 2 (3.7) |  |
| 8C | At my hospital, there is already a complete hospital policy on child maltreatment (+) | 9 (36.0) | 7 (28.0) | 9 (36.0) | 48 (55.8) | 15 (17.4) | 24 (27.9) | 26 (48.1) | 11 (20.4) | 18 (33.3) | 29 (53.7) | 10 (18.5) | 15 (27.8) |  |
| Statements regarding complete toolkit n=106/204 | | **N=24/46** | | | **N=81/158** | | | **N=52/98** | | | **N=51/98** | | |  |
| 1D | I think this toolkit leaves enough room for me to make my own conclusions (+) | 21 (87.5) | 2 (8.3) | 1 (4.2) | 64 (79.0) | 11 (13.6) | 6 (7.4) | 42 (80.8) | 5 (9.6) | 4 (7.7) | 40 (78.4) | 8 (15.7) | 3 (5.9) |  |
| 2D | I wish to know more about this toolkit before I decide to apply it (+) | 19 (79.2) | 3 (12.5) | 2 (8.3) | 59 (68.6) | 10 (12.3) | 13 (16.0) | 37 (71.2) | 4 (7.7) | 11 (21.2) | 38 (74.5) | 9 (17.6) | 4 (7.8) |  |
| 3D | I have problems changing my old routines (-) | 4 (16.7) | 1 (4.2) | 19 (79.2) | 6 (7.4) | 8 (9.9) | 67 (82.7) | 5 (9.6) | 5 (9.6) | 41 (78.8) | 4 (7.8) | 4 (7.8) | 43 (84.3) |  |
| 4D | I think other doctors or assistants will cooperate in applying this toolkit (+) | 19 (79.2) | 5 (20.8) | 0 (0.0) | 63 (77.8) | 10 (12.3) | 9 (11.1) | 39 (75.0) | 8 (15.4) | 5 (9.6) | 40 (78.4) | 7 (13.7) | 4 (7.8) |  |
| 5D | I think managers/directors will not cooperate in applying this toolkit (-) | 4 (16.7) | 4 (16.7) | 16 (66.7) | 16 (19.8) | 16 (19.8) | 49 (60.5) | **7 (13.5)** | **6 (11.5)** | **38 (73.1)** | **13 (25.5)** | **13 (25.5)** | **25 (49.0)** |  |
| 6D | I am willing to use this toolkit in the future (+) | 20 (83.3) | 3 (12.5) | 1 (4.2) | 68 (84.0) | 12 (14.8) | 1 (1.2) | 46 (88.5) | 4 (7.7) | 1 (1.9) | 39 (76.5) | 11 (21.6) | 1 (2.0) |  |
| 7D | I already work according to all parts of this toolkit (+) | 8 (33.3) | 4 (16.7) | 12 (50.0) | 39 (48.1) | 18 (22.2) | 24 (29.6) | 25 (48.1) | 9 (17.3) | 17 (32.7) | 22 (43.1) | 11 (21.6) | 18 (35.3) |  |
| ^ (+) indicates positive statement; (-) indicates negative statement. Bold figures indicate statistically significant differences between subgroups (p<0.05) leading to differences in barriers and/or facilitators, which are not significant after correction for multiple testing (Bonferroni p<0.002). | | | | | | | | | | | | | | |
